# Integrated control of dry root rot of chickpea caused by *Rhizoctoniabataticola* under the natural field condition

**DOI:** 10.1016/j.btre.2020.e00423

**Published:** 2020-01-20

**Authors:** Abdul Khaliq, Sartaj Alam, Irfan Ullah Khan, Dilawar Khan, Shakela Naz, Yaxin Zhang, Assar Ali Shah

**Affiliations:** a College of Plant Protection, Nanjing Agricultural University, Nanjing, 210095, PR China; b Department of Plant Pathology, Faculty of Crop Protection Sciences, The University of Agriculture, Peshawar, Pakistan; c Department of Biochemistry and Molecular Biology, College of Life Science, Nanjing University, Weigang, No. 1, Weigang 1, Nanjing, 210095, PR China; d College of History, Nanjing University, 163 Avenue, Xianlin, Nanjing, China; e Institute of Animal Science, Jiangsu Academy of Agricultural Sciences, Nanjing, 210014, PR China; f National Forage Breeding Innovation Base (JAAS), Nanjing 210014, PR China

**Keywords:** Aliette®, Bavistin®, Biomagic®, Chickpea, Fungus

## Abstract

The study investigated the effect of three fungicides Bavistin®, Aliette®, and Biomagic® at the rate of 2 g/L were tested *In vitro* and *In vivo* to control dry root rot and their impact on yield were evaluated at the rate of 4 g/L against *R. bataticola* while varieties used were Karak-1, Karak-2, Karak-3, and Sheenghar. Disease severity was recorded lowest (19 %) and highest yield (1467 Kg/ha), plant height (47.5 cm), number of pods/plant (15.0) and number of grain/pods (1.66) were recorded in cultivar Karak-1. Among the fungicides drugs, the Bavistin®, significantly reduced the disease severity (5 %) and Kg/ha), highest plant height (64.1 cm), highest grain yield (1488 Kg/ha), number of pods/plant (24.5) and number of grain/pods (2.25). It is concluded that the integrated use of Karak-1 and Bavistin®, fungicide drugs gave promising results for controlling dry root rot of chickpea, and increased plant height, number of pods/plant and number of grain/pods.

## Highlights

• *Bavistin*® was effective in controlling dry root rot of chickpea.
• Among the chickpea cultivars, Karak-1 was found to be resistant as compared to other cultivars.
• The highest plant (64.1 cm), highest grain yield (1488 Kg/ha) in the integrated use of Karak-1 and *Bavistin*®, fungicide.

## 1 Introduction

The chickpea yield has generally been low and erratic and one of the major limiting factors in obtaining higher yields Chickpea. The chickpea is susceptibility to various diseases and attacks of various fungal pathogens during the fields [1]. These cause enormous damage to the crop and thereby adversely affect the national economy. Overall, 169 pathogens attack chickpea on a worldwide basis which includes 66 fungi, 20 viruses, 3 bacteria, and 80 nematodes and mycoplasma [2]. Some of the serious diseases in order of their importance are dry root rot, wilt, wet root rot, and Ascochyta blight. Yield loss of 70 % by *Ascochyta rabieii* [3], 70 % by *R. bataticola* [4], 77–94 % by *Fusarium oxysporum f.sp. ciceri* [5], and 10–100 % by *Sclerotinia sclerotiorum* [6,7] has been previously reported. The problem of malnutrition can be minimized appreciably by controlling such devastating diseases in the country.

Dry root rot, caused by *Rhizoctonia bataticola* is a serious and widespread disease of chickpea. The causal organism is a soilborne necrotrophic fungus [8]. Pod setting and late-flowering are generally the stages where the plant is most vulnerable to dry root rot. Infected plants appear completely dried [1,3]. Destruction of lateral roots and extensive rotting are the symptoms commonly associated with the disease on rotten roots brittle and minute *clerotial* bodies appear mainly on the outer surface of the tape root and in the pith cavity [7,9].

Keeping in view the losses caused by dry root rot of chickpea, efforts should be made to effectively control the disease. Removal and destruction of diseased plant debris, crop rotation, rogueing of diseased plants, pod borer control, production of diseased free seed, proper storage of seeds and sowing of resistant varieties have been recommended for control of the disease [8,11].

The growers often argue for a disease control strategy that is rapid and effective. The use of fungicides is one such option [[9], [10], [11]]. In spite of their excessive use and abuse and build-up of pathogen resistance against these fungicides yet, control of plant-pathogen by chemicals is still the most popular and effective means of disease control. Such chemicals are readily available, relatively safe, easy to apply and less expensive. Additionally, some fungicides are eradicated and can help in getting rid of the pathogen that has already been established.

In view of the above facts, a need was felt to screen the available fungicides drugs in the market for their resistance build-up against the pathogen. Fungicides drugs showing promising results in terms of sensitivity as well as some new arrivals were evaluated further. The research is thus conducted to evaluate the effect of three different commercial fungicides drugs against *Rhizoctonia bataticola* chickpea to root rot under the natural field conditions.

## 2 Materials and methods

*In vitro* studies were carried out at the department of plant pathology, the University of Agriculture, Peshawar, Khyber Pakhtunkhwa, Pakistan. Three locally available fungicides Bavistin® (Thiophanate methyl), Aliette® (Fosetyl- Aluminium) and Biomagic® (*Bacillus subtilis*) were screened *in-vitro* in order to check their efficacy against *Rhizoctonia bataticola,* the causal organism of dry root rot of chickpea**.** Those showing promising results were further tested against the pathogen in a field trial. In the field experiment (*In-vivo*) was carried out at Agriculture Research Station, Ahmad Wala, Karak. Cultivars tested for resistance screening included four different varieties of chickpea such as Karak-1, Karak-2, Karak-3, and Sheenghar.

### 2.1 Isolation of different pathogen

The infected chickpea root and stem sample were collected from Agriculture Research Station Ahmad Wala, Karak for *in vitro* studies. The infected parts of chickpea roots and stems were cut into small pieces using sterilized scissors. The pieces were dipping in 0.1 % solution of mercuric chloride for 30 s and then rinsed three times in sterile distilled water (SDW) in order to remove the excess of disinfectant. The pieces were subsequently transferred to the center of Petri plates (9 cm diameter) containing the Potato Dextrose Agar (PDA) medium, under aseptic conditions in order to avoid contamination. The plates were incubated at 25 °C for one week. Colonies growing on PDA were subcultured on fresh PDA plates to get the pure culture of the pathogens. Identification of pathogen (*Rhizoctonia batatcola*) was identified on the basis of morphological examination and distinguishing characteristics using the mycological key of Aghakhani and Dubey [8,9,11].

### 2.2 *In vitro* efficacy of three different fungicides drugs against *R. Bataticola*

**Fig. 1:**
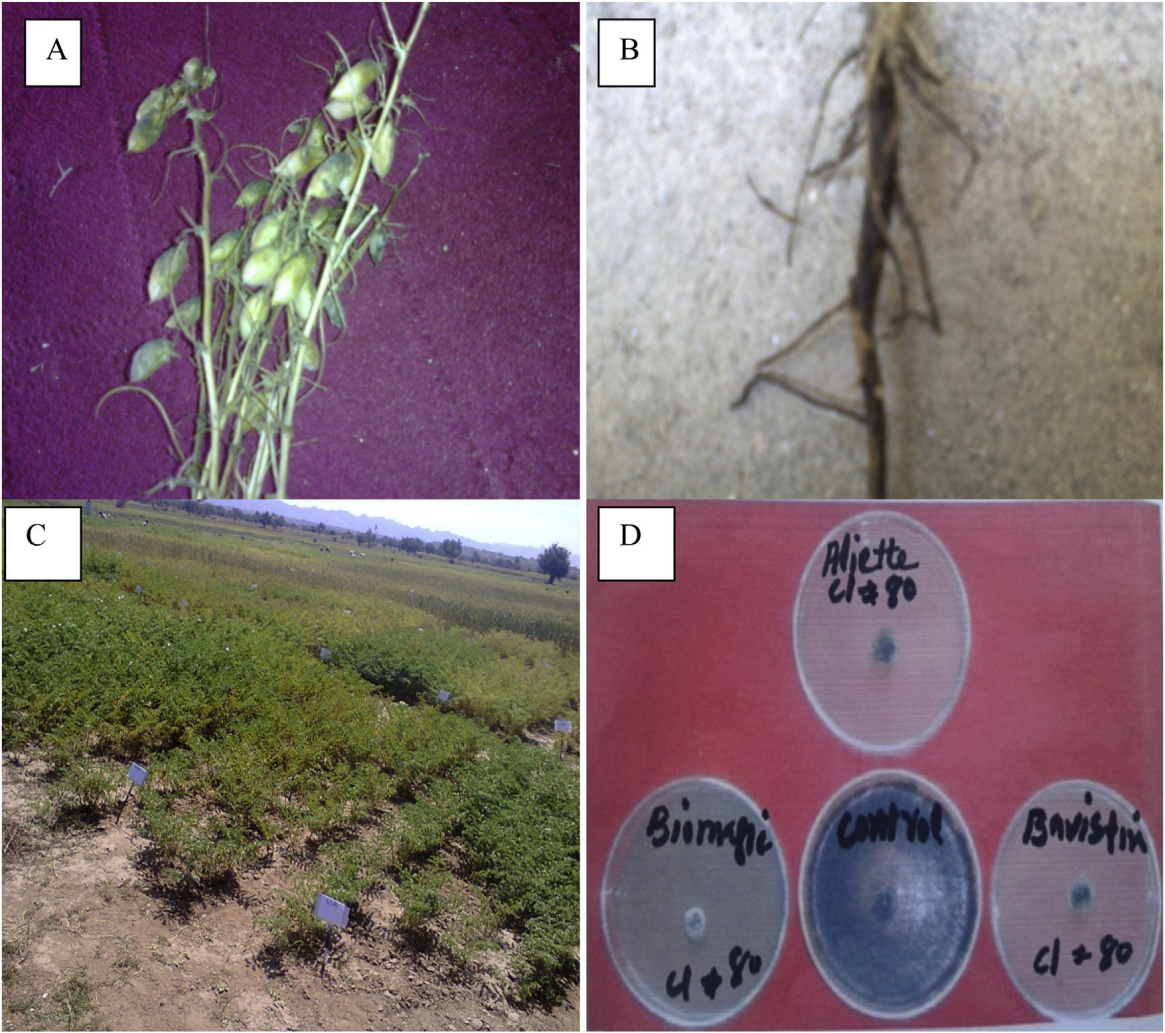
(A) general view of the chickpea grain (B) Typical dry root rot symptoms of chickpea (C) general view of the different varieties chickpea field collected from Ahmad Wala Karak, Khyber Pakhtunkhwa, Pakistan (D) *Rhizoctonia bataticola* growth of various fungicides.

*R. Bataticola* were cultured on Potato Dextrose Agar (PDA) medium under aseptic conditions containing 2 g/L of the three different fungicides (Aliette®, Bavistin®, and Biomagic) drugs. Streptomycin was added to the medium for the inhibition of bacterial growth before being poured into the ager plates. A 5 mm plug from the fresh culture of each isolate was a culture in the center of plates containing fungicide drugs amended medium. The un-amended plates served as control. The plates were then wrapped with cellophane and incubated at 25 °C for fungal growth. Data were recorded on the radial colony diameter of *R. bataticola* after 8 and 16 days (Fig. 1d). The radial colony growth of the pathogen was determined by measuring the colony diameter with the help of a ruler along two perpendicular axes and then taking the mean of the two measurements.

### 2.3 Field experiment (*in vivo* experiment)

In order to determine the best combination of cultivars and fungicides drugs against dry root rot. The field experiment was conducted at Ahmad Wala Research Station, Karak, Khyber Pakhtunkhwa, Pakistan. Four different varieties of chickpea including Karak 1, Karak 2, Karak 3, and Sheenghar, and each variety were used three different fungicides drugs e.g, Aliette®, Bavistin®, and Biomagic® were tested for their resistance against *R. bataticola*. Chickpeas were sown 1½ to 2 in. deep, spaced 3–6 inches apart with a row to row distance of 18–24 inches. Other agronomic practices were carried as per recommendation for all plots. The plot size for each treatment used was 1.2 m × 4 m. Each plot consisted of four rows. Fungicides drugs were applied by using a hand sprayer.

### 2.4 Disease severity

Disease severity was calculated as a percentage by visually observing the roots with symptoms (rotting or browning).

### 2.5 Days to 50 % flowering

Days to flowering was recorded from the date of sowing to the date when 50 % flowering had emerged and then the average was worked out.

### 2.6 Plant height (cm)

Plant height was recorded at the time of maturity by measuring the height of five randomly selected plants in each plot from the base of the plant to the top of the apical bud.

### 2.7 Number of pods plant

Numbers of pods plant^−1^ were recorded in a random sample of five plants from every three central rows which was then averaged.

### 2.8 Number of grain pod

The number of grain pod was recorded by selecting ten pods at random from each subplot. These pods were threshed and the number of grain pods was counted and then averaged.

### 2.9 Grain yield (kg ha^−1^)

Data on grain yield per sub- plot was recorded by harvesting the central four rows and their weight was taken by digital balance. Yield taken in grams was converted into Kg/ha by the following formula.

$$GrainyieldKgha)=\frac{\text{Grain yield}(\text{g})}{\text{R - R distance}(\text{m})\times \text{ No of rows x row length}(\text{m})}\times 10$$

### 2.10 Statistical analysis

Data were subjected by Analysis of Variance Test (ANOVA) to determine the significance of variation. LSD (5 %) was used for mean separation in case of significant differences between the treatments.

## 3 Results

### 3.1 *In vitro* screening of fungicides

**Fig. 2:**
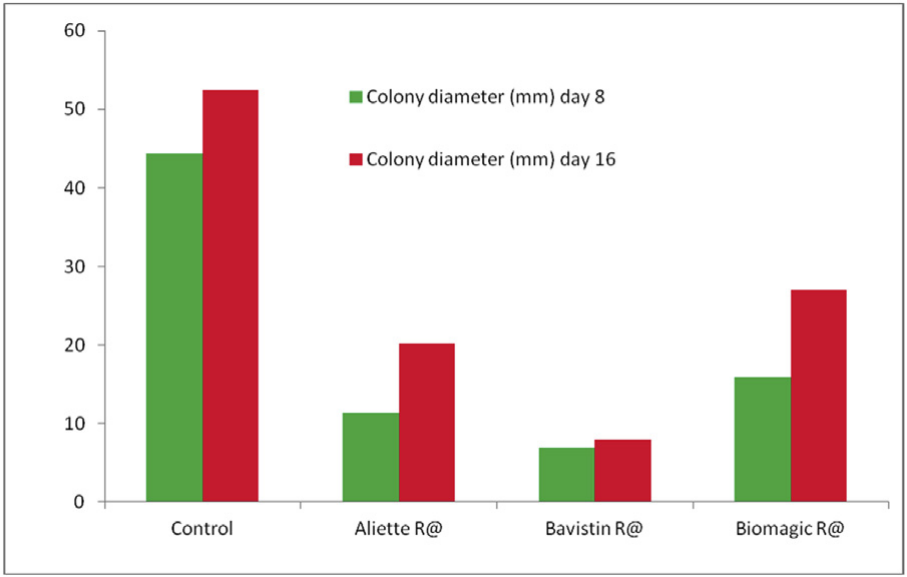
Effect of fungicides on colony diameter (mm) of *Rhizoctonia bataticola* on PDA amended with various fungicides after eight and sixteen days of incubation at 25 °C.

*In vitro* examination, the incubation period was 8 and 16 days at 25 °C, the Bavistin®, Aliette®, and Biomagic® fungicide drugs was significantly (P > 0.05) inhibited the growth of the pathogen as compared to control (Figs. 1d, and 2 ), but in these three fungicide drugs mostly effective drugs of Bavistin®.

### 3.2 Field studies (*in vivo* examination)

Based on the results, obtained from the *in-vitro* study, the fungicides were tested in combination with varieties in a field experiment.

### 3.3 Effect of fungicides on disease severity (%)

Effect of fungicides on disease severity (%) shown in Table 1. The interaction between varieties and fungicides was not significant (P = 0.99) for disease severity, but the main effects were significant (P = 0.00) among the groups and treatments. The disease severity among the cultivars and three different fungicides drugs, Bavistin®, (5 %), Aliette® (15 %) and Biomagic® (25 %) as compared to control (49 %) and the four different varieties of the chickpea, the lowest disease severity were recorded in Karak-1 (19 %), Karak-2 (28 %), Karak-3 (25 %) and Sheenghar (23 %).

**Table 1:** Effect of fungicides on disease severity (%) in different chickpea cultivars in a field experiment conducted at Ahmad Wala Karak.

Treatment Fungicides	Cultivars				Mean	Significant		
	Karak1	Karak2	Karak3	Sheenghar		F	V	F × V
Control	45	54	50	48	49a	*	*	*
Aliette®	12	19	17	15	15c	**	**	***
Bavistin®	1	10	7	5	5d	***	***	***
Biomagic®	21	30	27	24	25b	**	**	**
Mean	19d	28a	25b	23c		0.000	0.000	0.996

a, b, c, d - values bearing different letters in a row differ significantly (P < 0.05) the fungicides drugs were used 4 g/L distal water *in- vivo* experiment.

### 3.4 Effect of fungicides on days to 50 % flowering

Fungicides' effect on days to take a 50 % flowering is shown in Table 2. The interaction between varieties and fungicides was not significant (P = 0.53) for days to flowering. The cultivars and fungicides taken as main effects were statistically significant (P = 0.00) among the groups and treatments. The means were enhanced to flowering among the cultivars and three different fungicides drugs, Bavistin®, (98), Aliette® (85) and Biomagic® (72) as compared to control (60 %) and the four different varieties of the chickpea at 60 days planting to took the time to complete 50 % flowering, the lowest time were recorded in Karak-2 (73 days), and the highest time was recorded in Karak-1 (84 days) as compared to control.

**Table 2:** Effect of fungicides on days to 50 % flowering in different chickpea cultivars in a field experiment conducted at Ahmad Wala Karak.

Treatment Fungicides	Cultivars				Mean	Significant		
	Karak1	Karak2	Karak3	Sheenghar		F	V	F × V
Control	64	53	60	63	60d	*	*	*
Aliette®	90	81	82	87	85b	**	**	**
Bavistin®	105	92	95	98	98a	***	***	***
Biomagic®	77	67	70	76	72c	**	**	**
Mean	84a	73d	77c	81b		0.000	0.000	0.537

a, b, c, d - values bearing different letters in a row differ significantly (P < 0.05) the fungicides drugs were used 4 g/L distal water *in- vivo* experiment.

### 3.5 Effect of fungicides drugs on plant height (cm)

Effect of fungicides drugs on plant height (cm) shown in Table 3. The interaction between varieties and fungicides drugs was not significant (P = 0.20) for plant height. The significant differences among the fungicides were evident, with respect to plant height (P = 0.000) among the groups and treatments. The Bavistin® drugs were found to be effective in enhancing plant height (64.1 cm), Aliette® (49.0 cm) and Biomagic® (34.0 cm) and the tallest cultivars plants were Karak-1 (47.5 cm), Karak-3 (41.6 cm) Karak-2 (37.8 cm) and Sheenghar (42.2 cm).

**Table 3:** Effect of fungicides on plant height (cm) in different chickpea cultivars in a field experiment conducted at Ahmad Wala Karak.

Treatment Fungicides	Cultivars				Mean	Significant		
	Karak1	Karak2	Karak3	Sheenghar		F	V	F × V
Control	26.6	20.0	24.6	24.6	24.0d	*	*	*
Aliette®	54.6	43.0	47.0	51.6	49.0b	**	**	**
Bavistin®	69.6	59.3	63.0	64.6	64.1a	***	***	***
Biomagic®	39.6	29.0	32.0	36.0	34.0c	**	**	**
Mean	47.5a	37.8d	41.6c	44.2b		0.000	0.000	0.207

a, b, c, d - values bearing different letters in a row differ significantly (P < 0.05) the fungicides drugs were used 4 g/L distal water *in- vivo* experiment.

### 3.6 Effect of fungicides drugs on a number of pods plant

Data regarding a number of pods plant are shown in Table 4. The interaction between varieties and fungicides was not significant (P = 0.48) for a number of pods plant. Significant differences among the fungicides were evident, with respect to pods plant^−1^ (P = 0.00). Among the fungicides, Bavistin® was found to be effective in enhancing a number of pods plant. The highest number of pods plant was recorded for Bavistin® (24.5) followed by Aliette® (15.7) and Biomagic® (7.7) as compared to control (3.2). There were significant differences (P = 0.00) among the cultivars when pods plant was measured. Among the cultivars, the highest number of pods plant (15.0) was recorded on Karak-1 followed by Sheenghar (13.7), Karak-3 (12.0) and Karak-2 (10.5) as compared to control (24.0).

**Table 4:** Effect of fungicides on number of pods plant in different chickpea cultivars in a field experiment conducted at Ahmad Wala Karak.

Treatment Fungicides	Cultivars				Mean	Significant		
	Karak1	Karak2	Karak3	Sheenghar		F	V	F × V
Control	4.0	3.0	2.0	4.0	3.2d	*	*	*
Aliette®	18.0	13.0	15.0	17.0	15.7b	**	**	**
Bavistin®	28.0	20.0	24.0	26.0	24.5a	***	***	***
Biomagic®	10.0	6.0	7.0	8.0	7.7c	**	**	**
Mean	15.0a	10.5c	12.0bc	13.7ab		0.000	0.002	0.488

a, b, c, d - values bearing different letters in a row differ significantly (P < 0.05) the fungicides drugs were used 4 g/L distal water *in- vivo* experiment.

### 3.7 Effect of fungicides on number of grain pods

The effect of fungicides drugs on the number of grain per pods are shown in Table 5. The interaction between varieties and fungicides drugs was significantly (P = 0.04) greater number of grain per pods. When Bavistin® was sprayed on cultivars Karak-1 and Sheenghar then produced a significantly greater number of grain per pods as compared to Karak-2, Karak-3, and control. When Biomagic® was sprayed on cultivar Karak-3 then produced a significantly greater number of grain per pods as compared to Karak -1, Karak-2, Sheenghar and control groups. However, Aliette® drugs sprayed on four cultivars did not significantly affect and the greatest number of grain per pods was observed in Karak-1 (1.66) and Sheenghar (1.58) while the lowest number of grains per pods was observed in Karak-2 (1.16).

**Table 5:** Effect of fungicides on number of grain pods in different chickpea cultivars in a field experiment conducted at Ahmad Wala Karak.

Treatment Fungicides	Cultivars				Mean	Significant		
	Karak1	Karak2	Karak3	Sheenghar		F	V	F × V
Control	1.0ef	0.33g	0.66fg	1.oef	0.75c	*	*	*
Aliette ®	1.66cd	1.33de	1.66cd	1.66cd	1.58b	**	**	**
Bavistin®	2.66a	2.0bc	2.0bc	2.33ab	2.25a	***	***	***
Biomagic®	1.33de	1.0ef	2.0bc	1.0ef	1.33b	**	**	**
Mean	1.66a	1.16b	1.50a	1.58a		0.000	0.006	0.044

a, b, c, d - values bearing different letters in a row differ significantly (P < 0.05) the fungicides drugs were used 4 g/L distal water *in- vivo* experiment.

### 3.8 Effect of fungicide on grain yield (kg /ha)

The effect of fungicide drugs on grain yield Kg/ha is shown in Table 6. The interaction between fungicides and cultivars on grain yield Kg/ha was a significant effect (P = 0.01). The four cultivars did not differ significantly (P > 0.05) when Bavistin® and Aliette® drugs were sprayed. However, when Biomagic® was sprayed on Karak-2 gave significantly lower grain yield as compared to Karak-1, Karak-3, and Sheenghar and controls and the greatest number of grain yield Kg/ha was observed in Karak-1 (1467 Kg/ha), Sheenghar (1462 Kg/ha), while the lowest number of grains yield Kg/ha was recorded in Karak-2 (1455 Kg/ha). Bavistin® was found to be the most effective resulting in grain yield 1488 Kg/ha followed by Aliette® (1476 Kg/ha) while Biomagic ® was the least effective (1453 Kg/ha).

**Table 6:** Effect of fungicides on grain yield Kg/ha in different chickpea cultivars in a field experiment conducted at Ahmad Wala Karak.

Treatment Fungicides	Cultivars				Mean	Significant		
	Karak1	Karak2	Karak3	Sheenghar		F	V	F × V
Control	1432hi	1420i	1424i	1427i	1426d	*	*	*
Aliette®	1480bcd	1471de	1474cde	1477bcd	1476b	**	**	**
Bavistin®	1495a	1483abcd	1487abc	1490ab	1488a	***	***	***
Biomagic®	1461ef	1445gh	1451fg	1456fg	1453c	**	**	**
Mean	1467a	1455c	1459bc	1462ab		0.000	0.000	0.013

a, b, c, d, e, f, g, h, 1- values bearing different letters in a row differ significantly (P < 0.05) the fungicides drugs were used 4 g/L distal water *in- vivo* experiment.

## 4 Discussion

The pathogen *Rhizoctonia Bataticola* is a cosmopolitan fungus causing a number of diseases in different crops every year. The response of isolate of *R. bataticola,* collected from Agriculture Research Station Ahmad Wala, Karak was studied against different selected fungicides commercially used for the control of dry root rot of chickpea. An *in vitro* study was conducted on three different fungicides namely Bavistin®, Aliette® and Biomagic® against dry root rot pathogen. It was observed that among the fungicides Bavistin® and Aliette® successfully suppressed the growth of dry root rot fungus. Bavistin® has been reported to be systemic in its mode of action [7,12]. It seems that the fungicide was able to spread towards the canopy and root system well in time and therefore gave a wider coverage against the infection by the pathogen. Moreover, it contains Thiophanate Methyl as an active ingredient, which successfully inhibits the growth of many fungi including *R. bataticola* and *Botrytis cinerea* [7,12]. On the other hand, Biomagic® was found least effective probably because it was bio-fungicide which needs the proper environment for disease control. Bavistin® was found to be the most effective fungicide in reducing colony diameter after 16 days of incubation when compared to control where no fungicides were applied. According to Riaz et al. [1] found Bavistin® to be an effective fungicide in reducing colony diameter of *R. bataticola*. These results were in agreement with the finding of the present research. In previous studies, Singh et al. and Shah et al. [10,11] used Ridomil and sulphur against *R. bataticola* to suppress its growth under *in vitro* condition. Similarly, Toya and Patil [13] found that carbendazim alone and in combination with Thiram showed the best performance in reducing colony diameter of dry root rot fungus. Moreover, Peshrey et al. [14] have also reported similar results that carbendazim effectively inhibited the growth of dry root rot fungus. In our studies among the tested fungicides relatively poor results were obtained for bio-magic against the tested pathogen.

Among the fungicides drugs, Bavistin® and Aliette® gave promising results when tested in the field. The results are in agreement with [15] that tested nine fungicides against *R. bataticola in*-*vitro* and *in-vivo* in which Bavistin gave promising results. Among varieties, maximum disease severity was recorded on Karak -2, which also did not excel in yield and other agronomic characters. It could be due to continuous monoculture of this cultivar year after year which may have resulted in resistance break down. The high incidence of the disease in such a field might be due to the fact that the disease perpetuates through debris in the field. Cultivar Karak-1 was resistant as compared to other varieties which might be due to its genetic makeup and resistance to the disease. The results are in agreement with Mishra et al. [16] who tested 470 lines, of which KG86, KWR-4, and KWR-108 were found to be resistant. Similar findings were also reported by Chaturvedi and Dua [17] who reported 25 cultivars including K-50 and KPG-59 as resistant against dry root rot. Control through host resistance is arguably the best strategy for plant disease management. It is not only environmentally safe but also cost-effective.

The advantage that outweighs many of the disadvantages of fungicides is their rapid action in managing plant diseases which therefore makes them a panacea. However one should be careful in excessive use of fungicides to guard against resistance build-up in the pathogen as well as ground-water contamination. It is clear from the present studies that although the use of fungicides has resulted in the development of resistance against them, still it remains to be effective in suppressing the fungal growth [12,19]. Consequently, the farmers obtain promising yield. However, most of the fungal diseases are correlated with the environmental condition. In order to get a reduction in disease severity, either a single or a few timely sprays of fungicides are enough rather than indiscriminate use of fungicides which unfortunately effects untargeted beneficial microorganisms and also pollutes the environment [12,20]. Prevailing environmental conditions and management strategies are also significant factors in disease progression. A single application of *bavistin* at early stages after disease development may significantly reduce the diseases as compared to its application at later stages [12,15] Sometimes fungicides are combined together to enhance the fungicide spectrum and consequently inhibit the development of resistance among fungal pathogens [18,21,22].

## 5 Conclusions

*Bavistin*® was effective in controlling dry root rot of chickpea by reducing disease severity as well as improving yield. Among the chickpea cultivars, Karak-1 was found to be resistant as compared to other cultivars. The integrated use of Karak-1 and Bavistin gave promising results for controlling dry root rot of chickpea, cultivar Karak-1 in combination with fungicide Bavistin should be used by farmers for more effective control of the disease. Further studies should be conducted to optimize fungicide dose in combination with host resistance.

## Author contributions

Khaliq, A, and Alam, S conceived and designed the experiments; Khaliq, A; Khan, I, U; Naz, S and Zhang, Y performed the experiments; Khan, D; Shah A. A and Alam, S analyzed the data computationally; Khaliq, A and Shah, A. A wrote the manuscript.

## Funding

Not applicable.

## Declaration of Competing Interest

The authors consider that there is no controversy of interests.
